# Toxicity and Binding Profile of Lectins from the Genus *Canavalia* on Brine Shrimp

**DOI:** 10.1155/2013/154542

**Published:** 2013-11-27

**Authors:** Francisco Vassiliepe Sousa Arruda, Arthur Alves Melo, Mayron Alves Vasconcelos, Romulo Farias Carneiro, Ito Liberato Barroso-Neto, Suzete Roberta Silva, Francisco Nascimento Pereira-Junior, Celso Shiniti Nagano, Kyria Santiago Nascimento, Edson Holanda Teixeira, Silvana Saker-Sampaio, Benildo Sousa Cavada, Alexandre Holanda Sampaio

**Affiliations:** ^1^Integrated Laboratory of Biomolecules (LIBS-BioMol Group), Department of Pathology and Legal Medicine, Federal University of Ceará, 62042-280 Fortaleza, CE, Brazil; ^2^Laboratory of Biologically Actives Molecules, Department of Biochemistry and Molecular Biology, Federal University of Ceará, 60440-970 Fortaleza, CE, Brazil; ^3^Marine Biochemistry Laboratory, Fishing Engineering Department, Federal University of Ceará, 60440-970 Fortaleza, CE, Brazil

## Abstract

Lectins are sugar-binding proteins widely distributed in nature with many biological functions. Although many lectins have a remarkable biotechnological potential, some of them can be cytotoxic. Thus, the aim of this study was to assess the toxicity of five lectins, purified from seeds of different species of *Canavalia* genus. In order to determine the toxicity, assays with *Artemia* nauplii were performed. In addition, a fluorescence assay was carried out to evaluate the binding of lectins to *Artemia* nauplii. In order to verify the relationship between the structure of lectins and their cytotoxic effect, structural analysis was carried out to evaluate the volume of the carbohydrate recognition domain (CRD) of each lectin. The results showed that all lectins exhibited different toxicities and bound to a similar area in the digestive tract of *Artemia* nauplii. Concerning the structural analysis, differences in spatial arrangement and volume of CRD may explain the variation of the toxicity exhibited by each lectin. To this date, this is the first study that establishes a link between toxicity and structure of CRD from Diocleinae lectins.

## 1. Introduction

Lectins are sugar-binding proteins widely distributed in nature in organisms such as viruses, bacteria, fungi, plants, and animals [1]. Some of these proteins bind mono- and oligosaccharides specifically and reversibly but are devoid of catalytic activity and, in contrast to antibodies, are not products of an immune response [2]. Lectins have many biological functions, such as host defense, cell-cell interaction, glycoprotein folding, symbiosis, regulation of cell growth, apoptosis, fertilization, and other functions [3, 4]. Lectins purified from the Leguminosae family are the most extensively studied carbohydrate-binding proteins [5]. The subtribe Diocleinae has lectins that are multimeric structures composed of identical monomers of 25.5 kDa and exhibit a pH-dependent equilibrium between dimer and tetramer conformations. Diocleinae lectins also share the same carbohydrate recognition specificity for D-mannose and D-glucose, require divalent ions such as Ca^2+^ and Mn^2+^ to be biologically active, and contain a hydrophobic cavity that binds to phytohormones and other hydrophobic ligands. Despite their high similarity, these ConA-like lectins induce different responses in biological assays; for example, when tested for stimulation of human lymphocyte proliferation *in vitro*, ConBr had a higher proliferation index than ConA, possibly due to minor changes in binding specificities [6].

A broad range of lectins has been found to exhibit toxic and cytotoxic activities in several different assays. These effects include *in vitro* cytotoxicity in cultured lymphoid cells [7], tumor cells [8], and T cells [9] and *in vivo* toxicity after injection of the lectin into the peritoneal cavity of mice and lethality in the brine shrimp *Artemia sp*. [10]. In particular, the *Artemia* lethality test [11, 12] has been used successfully to determine the toxicity of bioactive compounds that have a variety of pharmacological activities, including anticancer agents, antivirals, insecticides, pesticides, and anti-HIV compounds [13–15]. The *Artemia* nauplii were used because they are highly sensitive. In addition, the cysts can be easily obtained, hatched, and stored at room temperature for several months without losing their viability [16]. In the present work, the toxicity of five lectins isolated from seeds of plants from the genus *Canavalia* was evaluated using the *Artemia* lethality test.

## 2. Materials and Methods

### 2.1. *Artemia* Nauplii Hatching

The *Artemia* cysts were hatched in artificial seawater at 28°C under constant lighting and strong aeration. The cysts were incubated in a polyethylene cylindroconical tube with 1 g cysts per liter of artificial seawater. This hatching condition simulates *Artemia's* natural environment, shallow seawater. After a period of 24 h, the aeration was halted, and the lighting was directed to the bottom of the hatching vessel. Due to the phototropic nature of the nauplii, they migrate in the direction of the light to the bottom of the tube, while the unhatched cysts float. The nauplii are then collected and used for bioassays.

### 2.2. Purification of Lectins

Lectins from *Canavalia ensiformis* (ConA), *C. brasiliensis* (ConBr), *C. boliviana* (ConBol), *C. grandiflora* (ConGF), and *C. maritima* (ConM) were extracted from air-dried ground seeds collected in Fortaleza, CE, Brazil, and defatted with n-hexane. The protein extract was obtained by continuous stirring with 0.9% NaCl (1 : 10 w/v) at 20°C for 4 h, followed by centrifugation at 10,000 g for 20 min at 4°C. The purification of the lectins was carried out using established methods [6]. The supernatant was submitted to affinity chromatography on a Sephadex G-50 column (5 **× **25 mm), equilibrated with 0.9% NaCl containing 5 mM CaCl_2_ and 5 mM MnCl_2_. Then the column was washed using the same buffer at a flow rate of 1 mL/min. The bound lectin was eluted with 0.1 M glycine, pH 2.6, dialyzed extensively against distilled water, and lyophilized. The purity of each lectin was monitored by SDS-PAGE, as described by Laemmli [17].

### 2.3. *Artemia* Lethality Test

Lectins were dissolved in artificial seawater at a concentration of 200 *μ*g/mL. The assay was performed boarding 24 well Linbro plates in which each well contained 10 *Artemia* nauplii in a final volume of 2 mL. Lectin solution was added to the wells at final concentrations of 12.5, 25, 50, or 100 *μ*g/mL. The experiments were performed in triplicate, and negative control wells contained 2 mL of artificial seawater with 10 *Artemia* nauplii. Another control group, which consisted of (bovine serum albumin) BSA at the same lectins concentration, was too included. After 24 h, the number of dead nauplii in each well was counted. From these data was calculated the percentage of death at each concentration and the LC_50_ value by probit analysis as described by Finney [18].

### 2.4. Blocking the ConBr CRD

A solution of 200 *μ*g/mL purified ConBr was incubated in artificial seawater containing 0.1 M *α*-methyl mannoside for 1 h at 37°C. That solution was then assayed in the *Artemia* lethality test at final concentrations of 12.5, 25, 50, or 100 *μ*g/mL ConBr. After 24 h, the number of dead nauplii was counted. The percentage of death and the LC_50_ value were calculated by probit analysis as described by Finney [18].

### 2.5. FITC-Labeled Lectin

FITC-labeled lectins were prepared in inhibition buffer (0.1 M D-mannose in 0.1 M carbonate-bicarbonate buffer, pH 9.0), conjugation buffer (0.1 M carbonate-bicarbonate buffer, pH 9.0), and washing buffer (phosphate-buffered saline: 0.01 M sodium phosphate buffer, 0.027 M KCl and 0.15 M NaCl, pH 7.4). Initially, the lectins were dissolved in inhibition buffer and incubated at 37°C for 1 h. Then, 250 *μ*L fluorescein isothiocyanate (FITC) (500 *μ*g/mL in conjugation buffer) was added dropwise. The solution was incubated for 2 h at room temperature under gentle stirring. Subsequently, unconjugated FITC was separated from FITC lectin by size exclusion chromatography using a Sephadex G-25 column previously equilibrated and eluted with washing buffer. The absorbance of all fractions was determined at 280 nm (protein) and 495 nm (FITC) to verify chromatographic efficiency. The FITC-labeled lectins were then dialyzed against 0.1 M acetic acid for 1 h to remove the blocker carbohydrate and extensively dialyzed against distilled water. The BSA was coupled to FITC following the same steps used to lectins, without the use of inhibition buffer.

### 2.6. Lectin Fluorescence Microscopy

Briefly, the shrimp were incubated with FITC-lectins or FITC-BSA (50 *μ*g/mL) and kept overnight. The shrimp were then washed 3 times in phosphate-buffered saline, placed on slides, and observed under a fluorescence microscope (Eclipse E200/epi-fluorescence, Nikon, Tokyo, Japan) equipped with a digital camera (DS-2Mv, Nikon, Tokyo, Japan). Images were acquired with NIS-Elements software version 2.3 (Nikon, Tokyo, Japan). The resolution of all acquired images was 5.0 Mpixel (Media Cybernetics, Silver Spring, MDI, USA).

### 2.7. Structural Analysis

The CRD volume of all lectins was calculated using the Q-SiteFinder program [19]. Briefly, crystal structures of *C. ensiformis* (PDB code: 1JBC), *C. brasiliensis* (PDB code: 3JU9), and *C. maritima* (PDB code: 2OW4) were obtained from Protein Data Bank (PDB) repository [20]. Atomic coordinates from *C. boliviana* (CBol) and *C. grandiflora* (ConGF) are in-house data and remain under publication. The structures were visualized using COOT [21] and PyMol [22].

## 3. Results and Discussion

**Figure 1:**
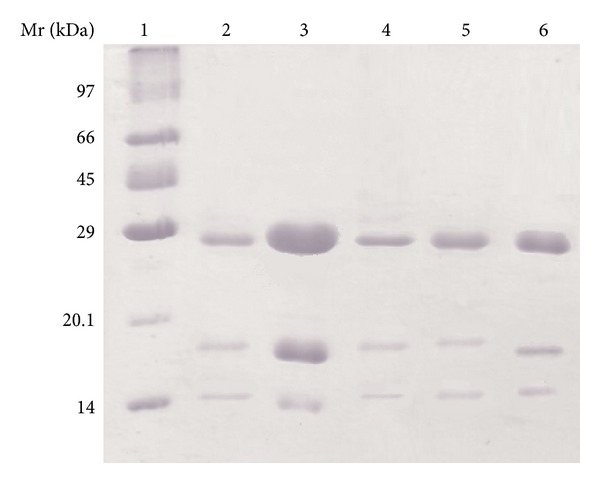
SDS-PAGE: (1) molecular mass markers (phosphorylase b, 97 kDa; bovine serum albumin, 66 kDa; ovalbumin, 45 kDa; carbonic anhydrase, 29 kDa; trypsin inhibitor, 20.1 kDa; and *α*-lactalbumin, 14 kDa); (2) ConBr (3) ConA, (4) ConM, (5) ConBol, and (6) ConGF.

The SDS-PAGE of ConBr, ConA, ConM, ConBol, and ConGF showed a pattern of subunits characteristic of the lectins from the Diocleinae subtribe. The proteins migrated through the electric field and were separated into three bands consisting of the full-length intact polypeptide chain (*α*-chain) and two fragments, *β* and *γ* (Figure 1).

*Canavalia* lectins exhibited a range of different toxicities toward *Artemia* nauplii. The LC_50_ values for ConA, ConBr, ConM, ConBol, and ConGF are shown in Table 1. The least toxic lectin in this assay was ConA, which had a LC_50_ of 376.48 *μ*g/mL. The most toxic was ConBr, which exhibited LC_50_ of 54.38 *μ*g/mL. When preincubated with *α*-methyl mannoside, the LC_50_ of ConBr increased from 54.38 *μ*g/mL to 337.75 *μ*g/mL (Table 1).

Toxicological studies have been conducted on all kinds of bioactive molecules and natural extracts [23, 24]. The *Artemia* lethality test has been used as a preliminary toxicity assay commonly performed before other bioassays that detect anticancer, antiviral, insecticide, pesticide, and anti-HIV activity; as such, the *Artemia* lethality test is very suitable for biotechnological purposes and the evaluation of bioactive compounds. Until now, only a small number of lectins have been evaluated in the *Artemia* lethality test. In most instances, lectins such as CSL-2 and PNL-2 have shown high toxicity [25–27]. In contrast, lectins such as SejaBL exhibit low toxicity [28].

It has been published elsewhere that to achieve more solubility of the lectins to perform this kind of biological tests the use of DMSO is suitable [14, 27]. Although DMSO has been reported to have low toxicity [29], this does not exclude the possibility that the *Artemia* toxicity is not affected by the addition of DMSO in these previous reports. Santos et al. [27] studying the toxicity of ConA-like lectins in the presence of DMSO found out a LC_50_ for ConBr of 15.4 *μ*g/mL. The present work obtained an LC_50_ of 54.38 *μ*g/mL in an assay that contained no DMSO (Table 1). Increased toxicity of ConBr in the presence of DMSO in the work by Santos et al. [27] may be due to a synergy between the two compounds. Ramos et al. [10] reported that a highly toxic lectin isolated from *Abrus pulchellus* had an LC_50_ of 3.5 *μ*g/mL without the addition of DMSO. The findings pointed out the fact that it is possible to carry on studies of lectin toxicity without the use of DMSO.

Preincubation of ConBr with *α*-methyl mannoside showed that the carbohydrate-recognition domain is involved in the toxic effect of the lectin on brine shrimp. When preincubated with the carbohydrate, the LC_50_ of ConBr increased from 54.38 *μ*g/mL to 337.75 *μ*g/mL (Table 1).

**Figure 2:**
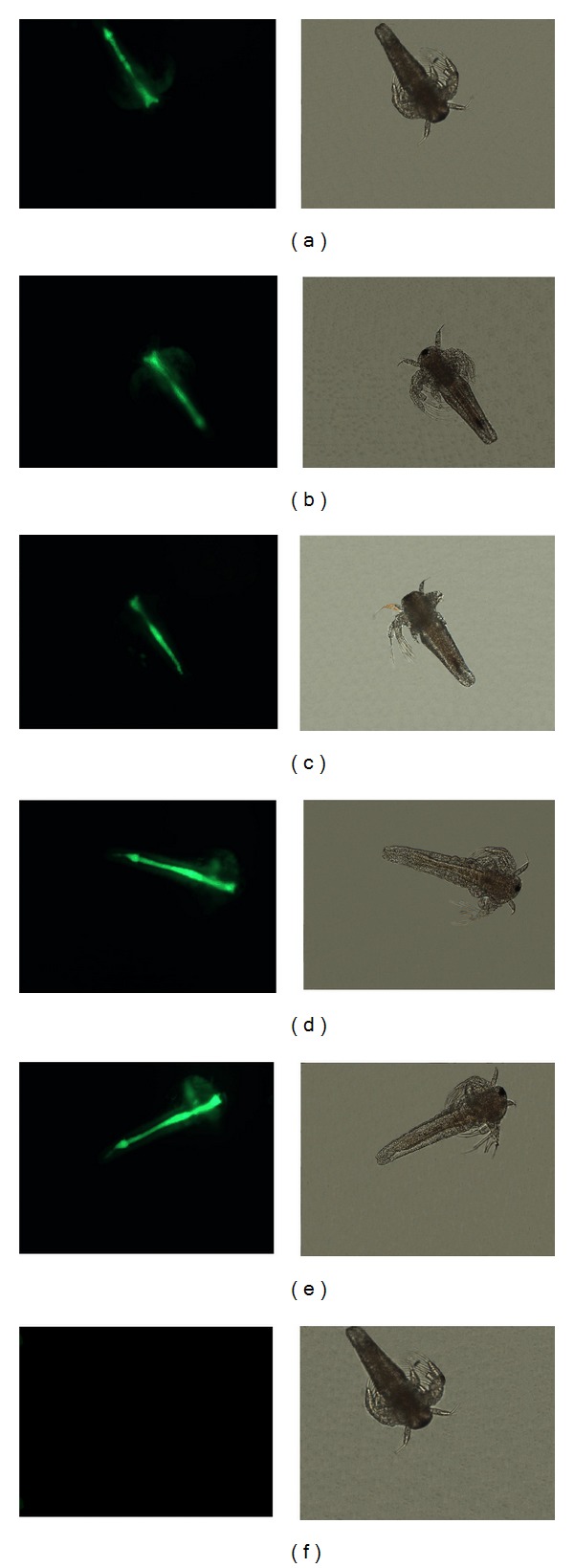
Binding of FITC-labeled lectins to *Artemia* nauplii detected by fluorescence microscopy. The green color indicates the presence of FITC-labeled lectins in the digestive tract of the animal. (a) FITC-ConBr; (b) FITC-ConA; (c) FITC-ConBol; (d) FITC-ConM; (e) FITC-ConGF; and (f) FITC-BSA. The same images were also acquired in bright field without fluorescence excitation.

Microscopy confirmed that lectins bound to *Artemia* nauplii (Figure 2). All lectins bound to a similar area of the organism and this area represents the digestive tract of the nauplii. The binding in this area was probably due to specific carbohydrate recognition, since the addition of *α*-methyl mannoside reduced the binding of the FITC-labeled lectins to the digestive tract of the nauplii (data not shown).

The exact mechanism by which lectins play its toxicity on *Artemia* is still unclear; however, fluorescence tests showed the presence of lectins in the digestive tract of *Artemia* nauplii, suggesting that the surface of the digestive tract is extensively glycosylated (Figure 2). Previous studies have shown that plant lectins have an affinity for the surface of gut epithelial cells of mammals and insects [30–32]. These lectins when attached to the surface of the gut epithelial cells may cause an antinutritional action, inhibiting the absorption of nutrients or occasionally causing toxic effects, leading to necrosis in the gut cells [30].

**Figure 3:**
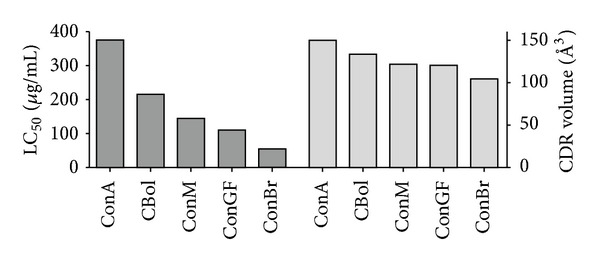
Relationship between CRD volumes and toxicity of the *Canavalia* lectins.

**Figure 4:**
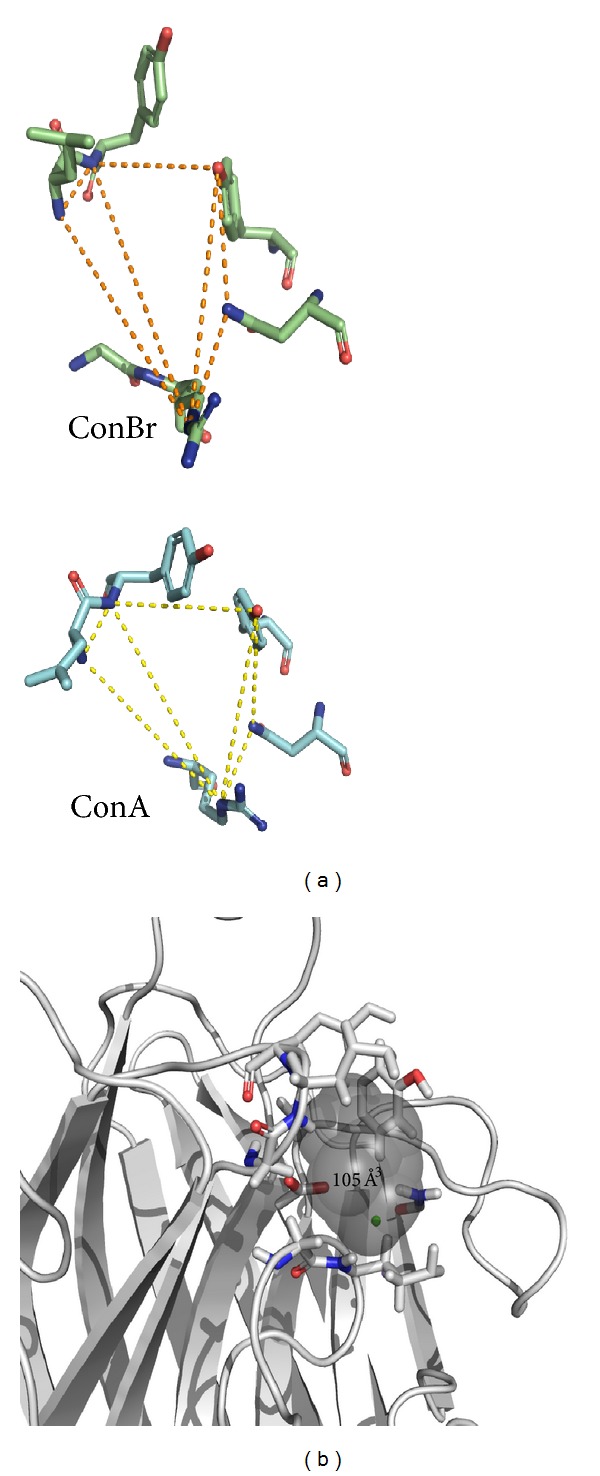
(a) Distances between residues comprising the CRD of ConBr (green) and ConA (blue). (b) ConBr structure with highlighted residues that comprise its CRD in sticks surrounding spheres, which comprise the volume of the CRD.

Despite the high structural similarity shared by *Canavalia* lectins, significant differences were seen when the toxicity was assessed on *Artemia* nauplii. Key distances between residues comprising the CRD will determine its shape and hence the volume of each domain (Figures 3 and 4). Such characteristics directly impact the carbohydrate recognition and elicitation of biological effects [33, 34].

Interestingly, a direct relationship between the CRD volume and the toxicity level of lectins on *Artemia* nauplii was achieved. Among the tested lectins, ConBr was the most toxic, with LC_50_ of 54.38 mg/mL. ConBr has a CRD volume of 105 Å^3^ (the smallest CRD volume among the tested lectins). On the other hand, ConA had an LC_50_ of 376.48 mg/mL and has a CRD volume of 151 Å^3^ (the largest volume among the tested lectins).

Thus, it was observed that the relationship between LC_50_ and CRD volume is directly proportional (Figure 3). That is, when the CRD volume is small the LC_50_ is low. Therefore, if the lectins are ordered according to LC_50_ and CRD volume, the following sequence is obtained: ConA (LC_50_ of 376.48 *μ*g/mL; CRD volume of 151 Å^3^) > ConBol (218.13 *μ*g/mL; 134 Å^3^) > ConM (146.55 *μ*g/mL; 122 Å^3^) > ConGF (110.51 *μ*g/mL; 121 Å^3^) > ConBr (54.38 *μ*g/mL; 105 Å^3^).

Despite the structural similarities shared between *Canavalia* lectins and hence in the specificities for carbohydrates, the term “ConA-like” is not suitable when the issue refers to biological activities. Cavada et al. [6] reported that minor changes in the overall structure of some lectins lead to different biological activities. These differences arise from changes in three major physicochemical parameters: binding specificity for complex carbohydrates, the pH-dependent oligomerization state (dimer-tetramer equilibrium), and the relative orientation of the carbohydrate-binding sites. The smallest CRD volume of ConBr among other *Canavalia* lectins has been shown to be the major reason of its lower activation of nitric oxide synthase in aortic endothelial cells [35]. In this study, the smallest CRD volume of ConBr proved to be the major reason of its high toxicity on brine shrimp.

Although *Canavalia* lectins are structurally similar, minor structural changes are the basis by which they differ from each other. Such modifications change the three-dimensional structure of these proteins (as in the CRD arrangement) and hence may cause significant differences in several biological activities. Thus, the structural analysis of lectins in response to a biological activity can provide valuable information concerning their mechanisms of action.

## 4. Conclusions

According to the data reported in this study, ConBr is the most toxic on brine shrimp, while ConA is the least toxic, and ConBol, ConM, and ConGF exhibit intermediate levels of toxicity. Concerning the experimental methodology, it is a new approach to establish the toxicity of lectins on *Artemia* nauplii. In addition, new insights were provided about the role of lectins structure and its effect on viability of brine shrimp, which probably will contribute in further studies.

## Figures and Tables

**Table 1:** LC_50_ and percentage of dead nauplii in different concentrations of lectins.

Lectins	Concentration (*μ*g/mL)	% Lethality 24 h	LC_50_ 24 h
ConA	12.5	20	376.48 (±72.25)
	25	20	
	50	30	
	100	27	
ConM	12.5	7	146.55 (±27.34)
	25	37	
	50	33	
	100	37	
ConBol	12.5	20	218.13 (±38.50)
	25	20	
	50	33	
	100	33	
ConBr	12.5	43	54.38 (±23.48)
	25	40	
	50	50	
	100	60	
ConGF	12.5	3	110.51 (±8.79)
	25	10	
	50	13	
	100	43	
ConBr + sugar	12.5	17	337.75 (±53.54)
	25	27	
	50	27	
	100	27	
